# Remarks on the Pathology, Causes and Treatment of Epidemic Cholera

**Published:** 1852-12

**Authors:** J. Lawrence Page


ART. III.—*Remarks on the Pathology, Causes and Treatment of Epidemic Cholera.* By J. LAWRENCE PAGE, M. D.

I take the liberty of inclosing to you an article on cholera, written by me about three years ago. My subsequent observations and reflections have strengthened the views therein expressed, and the recent report of Prof. Hamilton, of Buffalo, appears to confirm the opinion I have long entertained, that the causes of intermittent, remittent, and congestive (pernicious intermittent) fevers and cholera are identical.

If you think proper to give this article a place in your excellent Journal, with or without comment, you will oblige

Yours respectfully,

J. LAWRENCE PAGE.

RACINE, Wis., Nov. 1, 1852.

That *cholera* depends upon some miasmatic or aerial poison, is now pretty generally admitted. What that poison is we do not pretend to decide, nor does it signify. The nervous system is unquestionably primarily morbidly impressed; and whether by *koino* or *idio* miasmata is a question, the solution of which will aid us but little in arriving at a correct pathology, on which to predicate our therapeutics. The disease has generally prevailed more extensively and with greater violence in what are termed "malarial districts," where intermittent, remittent and congestive fevers prevail. The history of the disease from its first appearance on the banks of the Ganges, shows that its ravages have been greatest in *alluvial* countries, of *tertiary* formation, while those of *primary* formation have almost entirely escaped the epidemic. The great and fertile valley of the Mississippi being of the former character, has suffered severely, while amid the bleak and sterile hills and granite formations of New England, the disease cannot be said to have prevailed *epidemically*.

It would seem from this fact that the cause of cholera is somewhat analogous to that of the diseases just referred to, or at least that the epidemic is considerably modified thereby; and this opinion is strengthened by the analogy which exists between *Asiatic cholera* and congestive fever. Shrunken features, cold extremities, labored or hurried respiration, pulseless or nearly pulseless wrist, burning sensation at the pit of the stomach, great thirst, restlessness and serous dejections are symptoms common to both diseases. The experienced medical man cannot but be impressed with the resemblance of these two formidable diseases.

Oppression and consequent depression, a complete exhaustion of the nervous system, a retrograde motion of the *lacteals* and *lymphatics*, by which the fluids are poured out into the alimentary canal, constituting a sort of internal hæmorrhage, are the leading features of cholera.

What then are the indications of cure? Most clearly to relieve the oppression, call the circulation from the internal organs, determine to the surface, arrest the hæmorrhage and sustain the sinking energies of the system.

How can these be effected? The physicians of India resorted to *bleeding* to fulfill the first indications, and where this was effected early in the disease, it was oftentimes successful, but the objection to this practice is, the radical tendency to prostration which characterizes this disease, besides, in many instances it is quite impossible to draw blood. A safer, more effectual and less debilitating method of overcoming the congestion is the cold affusion, than which nothing has been found more efficacious in arousing the energies of the system in the collapse of *congestive* fever. It is now very generally adopted in the treatment of that disease at the South, and the profession are familiar with the mode of its application.

Its beneficial effects are well attested in cases of poisoning by opium and other narcotics, and in *asphyxia* from the inhalation of deleterious gases. Who has not felt the delightful shock of a shower-bath, and the agreeable sensation of reaction which follows? It is this shock, or repeated shocks, that arouse the paralyzed nervous system and bring on reaction, equalizing the circulation and restoring warmth to the surface and extremities. Who, then, would hesitate to resort to its use in *cholera asphyxia?* Certainly no one who has compared the phenomena of that disease with congestive fever, and has seen its marked beneficial effects in the latter disease, even where the case was apparently hopeless.

But this is no longer an experiment. It has been tried, and the testimony of Parks, Giaconimi, Goodlet and others, confirm its value as a therapeutical agent. I am satisfied that a more prompt and efficacious remedy does not exist wherewith to combat cholera, than the cold *douche*, or dashing cold water over the surface of the body. Its use, even where the surface is of an icy coldness is followed by heat of skin, elevation of the pulse, cessation of the cramps, and freedom of respiration. Here, then, we have the means of fulfilling the principal indications.

Opiates, astringents, and stimulants, to shut up the *waste gates*, and sustain the sinking energies of the system, fulfill the other indications. Caution, however, should be used in the administration of opiates that *narcosis* be not induced, which we have reason to fear has been the case in many instances. *Ice*, internally, is very grateful and doubtless very beneficial, and should be allowed *ad libitum*. Calomel, which has been so generally administered, is undoubtedly a valuable *adjuvant*, but it is in the *sequela* or fourth stage, or during convalescence (which is often expedited thereby) that we expect much benefit from this remedial agent.

To recapitulate — our theory is, that cholera *in every stage*, is a state of *congestion*, differing only in degree, analogous to the congestive fever of the malarial districts, and *somewhat* at least dependent on the same cause or causes. Our treatment, therefore, predicated on this view, cannot in many respects, differ from that which the experience of years has approved in that disease.

If these views be correct, we can easily reconcile the suggestion of several European and American physicians, that *quinine* is a preventive or antidote to cholera.

